# Krill Oil-In-Water Emulsion Protects against Lipopolysaccharide-Induced Proinflammatory Activation of Macrophages In Vitro

**DOI:** 10.3390/md15030074

**Published:** 2017-03-15

**Authors:** Gabriel A. Bonaterra, David Driscoll, Hans Schwarzbach, Ralf Kinscherf

**Affiliations:** 1 Department of Medical Cell Biology, Philipps-University Marburg, Robert-Koch-Straße 8, 35032 Marburg, Germany; hans.schwarzbach@uni-marburg.de (H.S.); ralf.kinscherf@staff.uni-marburg.de (R.K.); 2 Stable Solutions LLC, Easton Industrial Park, 19 Norfolk Avenue, South Easton, MA 02375, USA; d.driscoll@stablesolns.com; 3 Department of Medicine, University of Massachusetts Medical School, Worcester, MA 01655, USA

**Keywords:** krill oil-in-water emulsion, omega-3 fatty acids, phospholipids, LPS, cytokines, septic shock

## Abstract

Background: Parenteral nutrition is often a mandatory therapeutic strategy for cases of septicemia. Likewise, therapeutic application of anti-oxidants, anti-inflammatory therapy, and endotoxin lowering, by removal or inactivation, might be beneficial to ameliorate the systemic inflammatory response during the acute phases of critical illness. Concerning anti-inflammatory properties in this setting, omega-3 fatty acids of marine origin have been frequently described. This study investigated the anti-inflammatory and LPS-inactivating properties of krill oil (KO)-in-water emulsion in human macrophages in vitro. Materials and Methods: Differentiated THP-1 macrophages were activated using specific ultrapure-LPS that binds only on the toll-like receptor 4 (TLR4) in order to determine the inhibitory properties of the KO emulsion on the LPS-binding capacity, and the subsequent release of TNF-α. Results: KO emulsion inhibited the macrophage binding of LPS to the TLR4 by 50% (at 12.5 µg/mL) and 75% (at 25 µg/mL), whereas, at 50 µg/mL, completely abolished the LPS binding. Moreover, KO (12.5 µg/mL, 25 µg/mL, or 50 µg/mL) also inhibited (30%, 40%, or 75%, respectively) the TNF-α release after activation with 0.01 µg/mL LPS in comparison with LPS treatment alone. Conclusion: KO emulsion influences the LPS-induced pro-inflammatory activation of macrophages, possibly due to inactivation of the LPS binding capacity.

## 1. Introduction

Sepsis and septic shock due to Gram-negative pathogens are responsible for significant morbidity and mortality in human populations [1]. LPS binding to phagocytic cells stimulates the synthesis and release of cytokines, such as TNF-α, IL-1β, and IL-6 [2]. Cytokine secretion is an important component of host defense, but when overstimulation occurs, excessive cytokine secretion may lead to the systemic signs and symptoms of sepsis [3]. Exogenous or endogenous stimulation of biological factors that modulate the extent of binding of LPS to monocytes and macrophages may play a pivotal role in determining the outcome of endotoxin exposure [1]. In this context, serum factors that bind LPS may prevent macrophage activation [4]. In vitro and in vivo, HDL binds LPS and neutralizes it and the LPS-induced cytokine response is attenuated [5,6]. However, the phospholipid (PL) content, rather than the cholesterol content, correlates with the effectiveness of LPS neutralization [7]. Additionally, circulating levels of HDL are reduced in sepsis/septic shock, and this reduction is positively correlated with the severity of the illness [8], and decreased LDL levels (≤70 mg/dL) were associated with increased risks of sepsis [9]. In an optimal way, the substance used to neutralize the endotoxin effect during sepsis should be anti-oxidative, anti-inflammatory, and with endotoxin-binding capacity. In this context, omega-3 fatty acids (*n*-3 fatty acids) decrease the production of inflammatory eicosanoids, cytokines, reactive oxygen species (ROS) and adhesion molecules [10]. The key link between PUFAs and inflammation is that eicosanoids, which are among the primary mediators and regulators of inflammation during acute metabolic stress, are generated from 20-carbon PUFAs [10]. The three types of omega-3 fatty acids involved in human physiology are α-eicosapentaenoic acid (EPA) and docosahexaenoic acid (DHA), both of which are usually found in marine fish oils and linolenic acid (ALA), commonly found in plant oils. With respect to the precursor fatty acid ALA, in human it has poor bioconversion to the essential omega-3 fatty acids EPA and DHA and, therefore, it is an unreliable source for these bioactive fatty acids [11]. In this context, fish oil dietary supplements play a role of increasing the strategic importance in meeting daily requirements of essential nutrients [12].

Applications of intravenous lipid emulsions containing fish oil reduce the length of stay in hospital [13], as well as antibiotic use and mortality [14]. Moreover, fish oil with parenteral nutrition provided to septic intensive care patients increases plasma EPA, modifies inflammatory cytokine, improves gas exchange [15], and may exert profound influence on the status of immunocompetence and inflammation [16,17] The anti-inflammatory properties of marine omega-3 fatty acids have already been described [18,19]. In addition to triglycerides, marine *n*-3 fatty acids are also available in other forms, such as in crude krill oil (KO), which provides EPA and DHA, mainly in the form of PLs, and as ethyl esters of pharmaceutical grade, highly-concentrated preparations [18]. In this context, most recently, KO, which contains a significant portion of its *n*-3 LC-PUFA in PLs, is also increasingly found on the market, and is promoted as being of “higher efficacy” [12,20]. Additionally, most recently a new product category, derived from Antarctic krill (*Euphausia superba* Dana), has been brought onto the omega-3 market, characterized by a greater ease of absorption due to higher PL content [12,20].

KO comes from sustainable fisheries and is nearly at the beginning of a food chain, compared with fish sources that are more affected by environmental pollutants [12,20]. In addition, a higher fraction of omega-3 LC-PUFA is associated with PLs in KO, compared to triacylglycerol in fish oils, and this property may improve gastrointestinal absorption and bioavailability of omega-3 LC-PUFA [21]. KO contains PUFAs, including the bioactive EPA and DHA, (up to 35% *w*/*w* of the fatty acids profile), with up to 95% *w*/*w* PLs and up to 45% triglycerides [22]. According to these characteristics, we hypothesize that an injectable KO emulsion might in vitro exert anti-inflammatory properties from the presence of omega-3 fatty acids, and also bind endotoxin, thereby inhibiting LPS mediated effects, i.e., LPS is less able to stimulate and activate macrophages to release pro-inflammatory cytokines.

## 2. Results

### 2.1. Effect of KO Emulsion or LPS on the Viability of Differentiated Human THP-1 Macrophages

**Figure 1:**
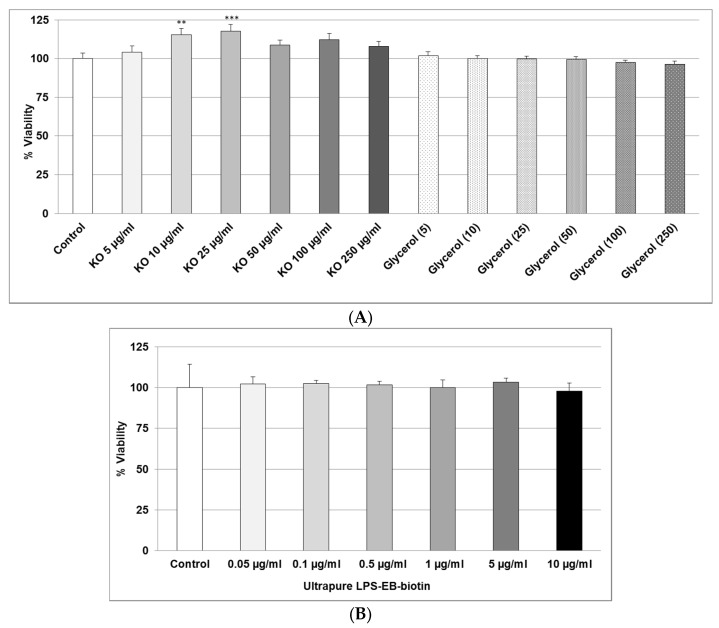
(**A**) Effects of 24 h treatment with KO emulsion or the glycerol vehicle (numbers in brackets indicate the volume of glycerol used in the corresponding KO concentration), on the viability of THP-1 macrophages. Values (in % viability of cells without treatment (control = 100%)) are given as the mean + SEM; ANOVA test, significance vs. negative control, ** *p* ≤ 0.01, *** *p* ≤ 0.001; *n* = 7 independent experiments; and (**B**) the effects of 4 h treatment with ultrapure LPS-EB-biotin on the viability of differentiated human THP-1 macrophages. Values (in % viability of cells without treatment (control = 100%)) are given as the mean + SEM; *n* = 4 independent experiments.

As shown in Figure 1A, we found that, after 24 h, treatment with 5–250 µg/mL KO did not display any cytotoxicity. Glycerol used as the vehicle was not cytotoxic (Figure 1A). Incubation of differentiated human THP-1 macrophages for 4 h with LPS did not show cytotoxicity (Figure 1B).

### 2.2. Effect of KO Emulsion on the LPS Binding

**Figure 2:**
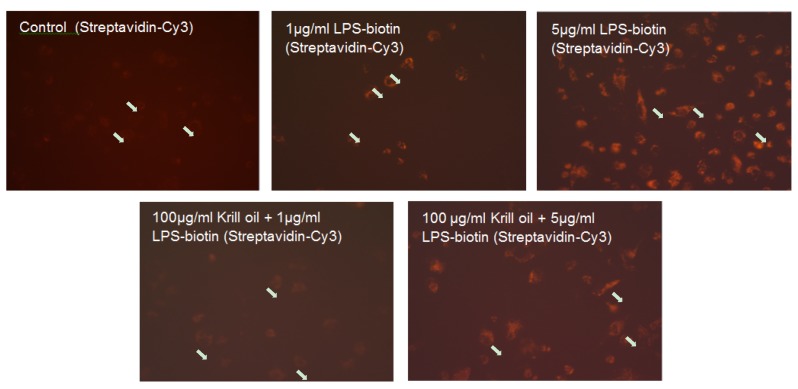
Effect of the KO emulsion on the LPS binding by differentiated human THP-1 macrophages. Photographs of the inhibitory effect of KO on the binding of ultrapure LPS-EB-biotin after 24 h incubation, was detected with streptavidin-Cy3-conjugated as fluorochrome. White arrows: nuclei. Magnification: 200×.

We used two binding assays to evaluate the interaction of LPS with macrophage-TLR4 and the inhibitory effect of KO. As shown in Figure 2, macrophages incubated 24 h with 1 µg or 5 µg/mL LPS-EB-biotin displays positive binding, detected by fluorescence (Figure 2), compared with controls without LPS.

**Figure 3:**
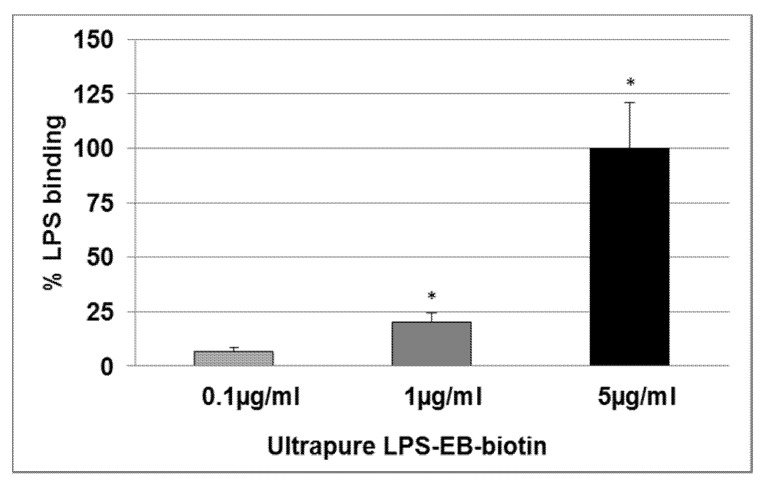
Effect of the KO emulsion on the LPS binding on macrophage-TLR4. Ultrapure LPS-EB-biotin binding assay was performed by using PMA-differentiated THP-1 macrophages after 3 h incubation, including streptavidin-HRP-OPD system for detection and colorimetric quantification. Values (binding relative to negative control (value = 1) without LPS-EB-biotin) are given as the mean + SEM; ANOVA test, significance vs. negative control without LPS, * *p* ≤ 0.05; *n* = 4 independent experiments.

LPS (1 µg/mL) pre-incubated with KO (100 µg/mL) inhibited the binding, but not when using LPS at a concentration of 5 µg/mL (Figure 2). The LPS binding was increased at 0.1 µg/mL (6.5%), 1 µg/mL (20.3%, *p* ≤ 0.05), and 5 µg/mL (100%, *p* ≤ 0.05) when compared with the negative control (Figure 3).

**Figure 4:**
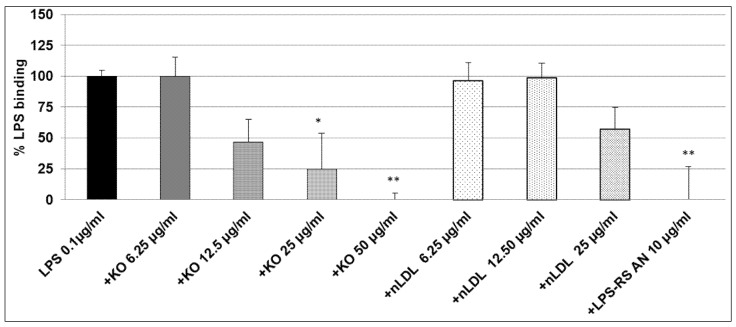
Ultrapure LPS-EB-biotin binding assay on macrophage-TLR4. LPS-EB-biotin (0.1 µg/mL) was co-incubated overnight with different concentrations of KO emulsion or LPS antagonist (LPS-RS AN), added to differentiated THP-1 macrophages (3 h). LPS binding was spectrophotometrically quantified using streptavidin-HRP-OPD system. Values (in % binding relative to positive control (100% binding) with LPS-EB-biotin) are given as the mean + SEM; ANOVA test, significance vs. positive control (=LPS-EB-Biotin), * *p* ≤ 0.05, ** *p* ≤ 0.01; *n* = 6 independent experiments.

After co-incubation of 0.1 µg/mL LPS-EB with KO, the macrophages were treated for 3 h with different concentrations of LPS + KO: at 12.5 µg/mL LPS + KO, LPS-binding decreased by 50% (not significant); at 25 µg/mL LPS + KO LPS binding significantly decreased by 75% (*p* ≤ 0.05) and, at 50 µg/mL LPS + KO, it was abolished (*p* ≤ 0.01), as shown in Figure 4.

**Figure 5:**
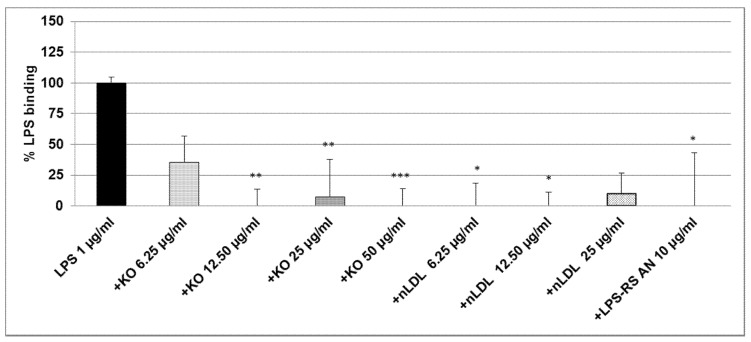
Ultrapure LPS-EB-biotin binding assay on macrophage-TLR4. LPS-EB-biotin (1 µg/mL) was co-incubated overnight together with different concentrations of KO emulsion or 100 µg/mL LPS antagonist (LPS-RS AN) added to differentiated human THP-1 macrophages (3 h). LPS binding was spectrophotometrically quantified using streptavidin-HRP-OPD system. Values (in % binding relative to positive control (100% binding) with LPS-EB-biotin) are given as the mean + SEM; ANOVA test, significance vs. positive control (=LPS-EB-Biotin), * *p* ≤ 0.05, ** *p* ≤ 0.01, *** *p* ≤ 0.001; *n* = 6 independent experiments using four wells per treatment and experiment.

As the LPS-binding control, 25 µg/mL nLDL showed a similar effect as KO at 12.5 µg/mL, however, this was not significant. LPS-RS-AN antagonist significantly (*p* ≤ 0.01) prevented the binding of LPS-biotin and, thus, mimicked the effect of KO (Figure 4). A total and significant inhibition of the binding was obtained when 1 µg/mL LPS-EB was co-incubated with 6.25, 12.5 µg/mL (*p* ≤ 0.01), 25 µg/mL (*p* ≤ 0.01), or 50 µg/mL (*p* ≤ 0.001) KO when compared with the control. Furthermore, LDL showed a similar inhibition pattern as KO. Additionally, the LPS-RS antagonist completely inhibited the LPS binding on the TLR4 (Figure 5). Moreover, we have performed this experiment using 5 µg/mL LPS-EB-biotin, however, without a statistically significant difference between LPS/KO co-incubation and LPS alone (data not shown).

### 2.3. Effect of KO Emulsion on TNF-α Release of Differentiated Human THP-1 Macrophages Stimulated with LPS-EB

**Figure 6:**
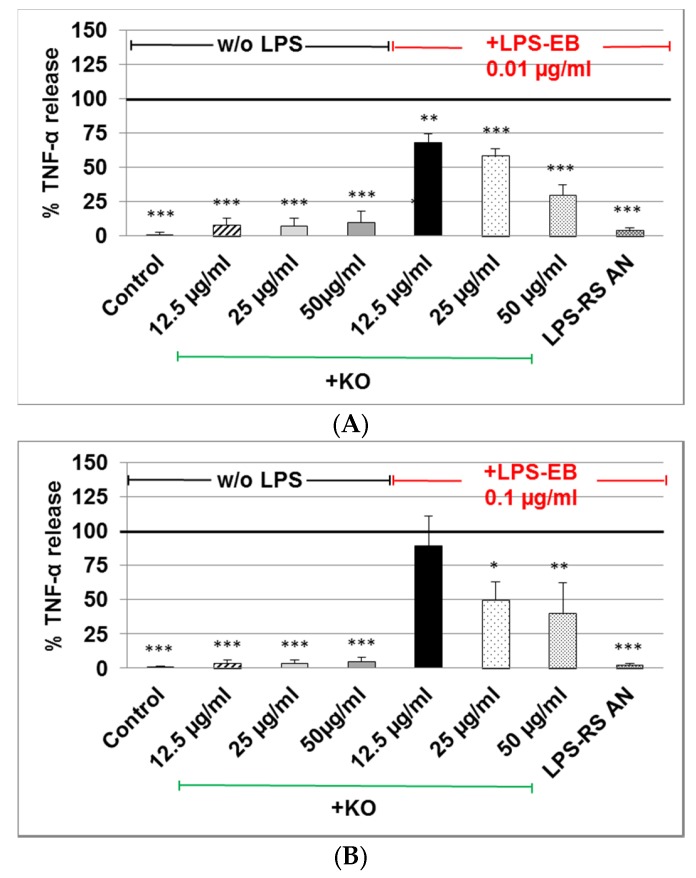
Analyses (by ELISA) of the inhibitory effect of KO emulsion on the TNF-α release by LPS-EB differentiated human THP-1 macrophages. (**A**) LPS-EB 0.01 µg/mL, or (**B**) 0.1 µg/mL was co-incubated overnight together with different concentrations of KO or 10 µg/mL LPS antagonist (LPS-RS-AN) and afterwards for 3 h with the differentiated THP-1 macrophages. Values (in %TNF-α release relative to positive (LPS-EB) control (=100% release)) are given as the mean + SEM; ANOVA test, significance vs. positive control (=LPS-EB), * *p* ≤ 0.05, ** *p* ≤ 0.01, *** *p* ≤ 0.001; *n* = 3–5 independent experiments.

The TNF-α release in cell supernatants of differentiated human THP-1 macrophages treated with LPS-EB (pre-incubated with KO) was markedly reduced when 0.01 µg/mL or 0.1 µg/mL LPS was used. A significant inhibition of the TNF-α release was obtained after incubation of 0.01 µg/mL LPS with 12.5 µg/mL (−30%, *p* ≤ 0.01), 25 µg/mL (−40%, *p* ≤ 0.001) or 50 µg/mL (−75%, *p* ≤ 0.001) KO in comparison with the control LPS (Figure 6A).

Treatment with 0.1 µg/mL LPS pre-incubated with 25 µg/mL or 50 µg/mL KO showed a 50% (*p* ≤ 0.05) or 60% (*p* ≤ 0.01) inhibition in comparison with control LPS (Figure 6B). Treatment with KO alone had no effect on the TNF-α production (Figure 6B). Incubation with antagonist LPS-RS abolished the TNF-α release (Figure 6B).

## 3. Discussion

Sepsis has been defined as a systemic immune activation in patients with infection, characterized by low rates of survival [23]. Bacterial endotoxemia is considered one of the major causes of sepsis, resulting from the release of LPS by microorganisms, e.g., within the colon, that translocate across a compromised intestinal wall [17,24]. In this context, we performed in vitro experiments to test the efficacy of a KO emulsion against endotoxin-triggered pro-inflammatory effects.

Death rates in patients caused by sepsis reach about 30%–80% [25], especially in oncologic patients [26]. Sepsis-associated organ failure and death result from an overwhelming inflammatory immune response that culminates in a generalized autodestructive process [27] and development of multi-organ dysfunction syndrome, or MODS [28]. Under normal physiological conditions, in which ROS levels are controlled by endogenous anti-oxidant systems, sepsis induces an imbalance between pro- and antioxidant systems, which leads to oxidative stress [29]. Moreover, long-chain *n*-3 PUFAs decrease the production of inflammatory mediators (eicosanoids, cytokines, and ROS) [22,30]. Once incorporated into cell membranes, a key step to exert cytoprotection, *n*-3-FAs dramatically modulate the body’s response to inflammation, oxidative stress, ischemia, and immune function through several downstream bioactive mediators, (e.g., cytokines, prostaglandins, thromboxanes, leukotrienes, resolvins, protectins, etc.) [22]. The combination of “cytoprotective excipients” [31], together with nutritional, anti-oxidant, and anti-inflammatory properties, make the omega-3 therapy a successful candidate for the management of sepsis in patients under intensive care. By using EPA or fish oil, inhibition of endotoxin-induced TNF-α production by monocytes [32], may exert effects on both, the generation of inflammatory mediators, and on the resolution of inflammatory processes [15]. These observations suggest direct effects of long-chain *n*-3 PUFA on inflammatory gene expression via inhibition of activation of the transcription factor NF-κB [33]. Several studies in healthy human volunteers involving supplementation of the diet with fish oil have demonstrated decreased production of TNF-α, IL-1β, and IL-6 after endotoxin-stimulation of monocytes or mononuclear cells [34,35]. The benefits of fish oil in animal models of experimental endotoxemia have been clearly demonstrated when dietary fish oil or fish oil infused intravenously enhances the survival of guinea pigs after intraperitoneal endotoxin injection [36]. In this context, strategies to attenuate the immune response and prevent organ failure could help patients with sepsis or septic shock. Given that ultrapure LPS-EB specifically binds to TLR4 and the LPS antagonist LPS-RS, leading to an almost complete suppression of the TNF-α release, confirms the specific binding of LPS-EB to TLR4. The same effect observed with KO, can be explained by an inactivation of LPS during the co-incubation with KO or an inhibition of the binding to TLR4. Nevertheless, the present study clearly demonstrates that KO inhibited the production of TNF-α after stimulation with LPS. Available data have confirmed an effective reduction in the LPS level in the patients’ blood after this procedure [37], and could effectively eliminate a wide range of the factors as LPS, cytokines, etc., from peripheral blood.

Our findings suggest that KO has properties to inactivate and bind LPS, leading to the inhibition of activation of macrophages. In this context, we found a reduction of LPS binding capacity on differentiated human THP-1 macrophages after 3 h treatment with LPS that has been co-incubated (24 h) with 100 µg/mL KO (Figure 2), by reduction of the fluorescence intensity at a concentration of 1 µg/mL LPS (Figure 2). These data support studies showing less LPS internalization into murine macrophages after 24 h treatment, when LPS was pre-exposed to high-density lipoprotein (HDL) [4]. In agreement with these studies, we used human LDL as binding control and we found that LDL reduced the binding of LPS on macrophages (Figure 4 and Figure 5). Moreover, KO significantly reduced the LPS-binding on macrophages over a range of LPS concentrations when both had been previously co-incubated and, afterwards, were applied to the macrophages. Human serum or LDL inactivate endotoxins and inhibit the IL-1β release in LPS-activated monocytes [38]. Consequently, this observed KO characteristic may be a sign of a possible LPS adsorption, inactivation, or hiding of the binding sites. Using the LPS antagonist *Rhodobacter sphaeroides*, which binds to the TLR4 but does not induce TLR4 signaling, we can confirm the specificity of the activation (Figure 4 and Figure 5). After co-incubation (24 h) of LPS and KO (12.5, 25 or 50 µg/mL), LPS lost its pro-inflammatory capacity and inhibited the TNF-α release. KO alone or LPS antagonist did not affect the TNF-α production, and confirmed that KO has beneficial, and not detrimental, effects. Therefore, our results impressively indicate that KO can efficiently prevent macrophages from being activated by LPS, including suppression of negative consequences. like the release of pro-inflammatory cytokines, such as TNF-α.

The effects we have observed with a KO emulsion are likely the result of two main mechanisms of action. First, the phopsholipids present in KO bind and neutralize LPS endotoxin. This effect has been shown previously in a study of normal human volunteers receiving an intravenous dose of *Escherichia coli* endotoxin during a 6-h PL infusion, and attenuation of the clinical and laboratory responses were directly related to PL levels in the bloodstream [39]. However, when this formulation was tested in the critical care setting in patients with severe Gram-negative sepsis, the high dose arm (1350 mg/kg by continuous infusion) had to be stopped because of the increased incidence of life-threatening significant adverse events and obvious futilty to show a survival advantage [40]. The PL emulsion used in this investigation consisted of 92.5% soy-based, PL, and 7.5% soy triglycerides. Although this formulation contained PLs capable of binding endotoxin, the dosing was probably excessive. Additionally, the fatty acid profile of soybean oil-derived triacylglycerols and phosphoglycerides mainly contain pro-inflammatory omega-6 fatty acids (i.e., linoleic acid). In contrast, the KO-based PL emulsion in the present study contained both the necessary PL to bind the endotoxin, plus the anti-inflammatory omega-3 fatty acids to modulate eicosanoid metabolism, and they clearly exhibit positive synergistic effects.

Finally, the KO emulsion used in this study was a crude formulation made from an unrefined natural source that is widely found in oral supplements and, thus, would be unsuitable for intravenous administration. Therefore, our study provided an indication of a proof-of-concept with this phospholipid-omega-3 combination in a cell culture model. We clearly recognize that for such a product to be a potentially viable injectable emulsion in the clinical setting, the crude KO would need to undergo refinement steps (similar to that applied to fish oil) [41] to concentrate the PL in amounts and levels of purity similar to currently available and widely used egg PL which, for example, contain at least 80% phosphatides (versus ~40%, and about equal amounts of triglycerides). In addition, since the triglycerides present in crude KO are essentially devoid of omega-3 fatty acids, the ideal injectable product would also include fish oil triglcyerides enriched with omega-3 fatty acids for maximal therapeutic efficacy.

We conclude that KO emulsion inhibits the LPS-binding on macrophage-TLR4 and, thus, the TNF-α release induced by LPS in vitro. The addition of omega-3 fatty acids potentiates the therapeutic actions by reducing the intensity of the systemic inflammatory response. These properties may be beneficial for patients under intensive care with septicemia.

## 4. Materials and Methods

### 4.1. Krill Oil-In-Water Emulsion

Three separate batches of a 5% KO-in-water emulsion were aseptically prepared in the laboratory and sterilized prior to use. This was done to be sure the emulsions could be successfully made using a crude source of KO, since a pharmaceutical-quality grade, suitable for parenteral administration, does not exist. The mean droplet size of all the emulsions was approximately 190 nm and, thus, they were considered pharmaceutically equivalent. The composition of the final emulsions are shown in Table 1.

### 4.2. Cells and Culture Conditions

The in vitro experiments were performed using the THP-1 (human acute monocytic leukemia) cell line (DSMZ GmbH, Braunschweig, Germany), cultured in 90% RPMI-1640 (PAA GmbH, Cölbe, Germany), 10% FBS (PAA GmbH); 100 U/mL penicillin; and 0.1 mg/mL streptomycin (PAA GmbH). All experiments were carried out in medium with 10% FBS.

### 4.3. Determination of LPS and KO Emulsion Cytotoxicity

THP-1 (5 × 10^4^) cells were seeded in 96-well plates (BD Falcon™, Becton Dickinson GmbH, Heidelberg, Germany). After differentiation with 0.1 µg/mL phorbol-12-myristate-13-acetate (PMA) Sigma-Aldrich, St. Louis, MO, USA), the medium was changed and the macrophages treated with 0.05 µg/mL-10 µg/mL ultrapure biotinylated lipopolysaccharide (LPS-EB) from *E. coli* O111:B4 (Cayla-InvivoGen Europe, Toulouse, France), which is recognized only by toll-like receptor-4 (TLR4); KO 5 µg/mL to 250 µg/mL, or glycerol. After 24 h KO or 4 h LPS treatment, viability was assessed using PrestoBlue™ reagent (Invitrogen-Life Technologies GmbH, Darmstadt, Germany). After 1 h the optical density (OD) was measured at 570 nm/600 nm with a SUNRISE ELISA-reader (Tecan, Salzburg, Austria). Results are expressed as the % of viability/survival (OD570 nm/600 nm of samples × 100/OD570 nm/600 nm of control without substances).

### 4.4. Determination of the LPS Binding on Differentiated THP-1 Macrophages, Effect of the Treatment with KO Emulsion, Detected by Fluorescence Microscopy

THP-1 (5 × 10^4^) cells were seeded in 100 µL medium/well in 96-well plates (BD Falcon™). After eight days of differentiation into macrophages using 0.1 µg/mL PMA, the medium was changed and the macrophages were treated with 1 µg/mL or 5 µg/mL ultrapure LPS-EB-biotin alone or with KO. After 3 h the cells were fixed with 1% paraformaldehyde (PFA/PBS) for 20 min and incubated afterwards with streptavidin-Cy3 (Dianova, Hamburg, Germany). Digitalized images were obtained using an inverted microscope Eclipse-TS100 (Nikon GmbH, Düsseldorf, Germany) and an AxioCamMRc/AxioVision digital imaging system (Carl Zeiss GmbH, Jena, Germany).

### 4.5. Determination of the LPS Binding on Differentiated THP-1 Macrophages Detected by Spectrophotometry

THP-1 (5 × 10^4^) cells were seeded as described above and treated with 0.1 µg/mL, 1 µg/mL, or 5 µg/mL ultrapure LPS-EB-biotin. After 3 h the macrophages were fixed with 1% PFA/PBS for 20 min, then incubated with streptavidin-biotinylated horseradish peroxidase (HRP-streptavidin-biotin) complex Amersham (GE Healthcare Europe GmbH, Freiburg, Germany). Subsequently, the macrophages were incubated with 50 µL peroxidase substrate Sigma Fast™ (OPD) (Sigma-Aldrich, St. Louis, MO, USA) for 30 min at RT. The reaction was stopped with 25 µL 3N HCl, and the absorbance was measured at 490 nm/655 nm. Afterwards, the cells were stained with crystal violet solution (0.04% paraformaldehyde crystal violet in 4% (*v*/*v*)). The OPD absorbance was normalized against the crystal violet absorbance measured at 595 nm/660 nm.

### 4.6. Inhibitory Effects of the KO Emulsion on the LPS Binding Capacity

PMA-differentiated THP-1 macrophages were treated for 3 h with the LPS-EB-biotin and KO mixture (both previously incubated together at 4 °C overnight), afterwards the cells were fixed with 1% PFA-PBS for 20 min, washed with PBS, incubated with streptavidin-biotin-complex-HRP (Amersham, GE Healthcare), afterwards with OPD and measured as described above. The positive control was performed by incubation of macrophages with ultrapure LPS-EB-biotin alone and the negative controls incubated only with medium or with KO. In humans, low-density lipoproteins (LDLs) may bind LPS and inactivate it; we used native LDL (nLDL, HoelzelDiagnostika GmbH, Cologne, Germany) as a control to compare the adsorption properties of KO [38]. The antagonist LPS from Rhodobactersphaeroides (LPS-RS) was used as control of the TLR4 specific binding by competitive inhibition at 100-fold excess of the agonist LPS-EB.

### 4.7. Effects of the KO Emulsion on TNF-α Release

The release of TNF-α was determined using ELISA. Therefore, 5 × 10^5^ THP-1 cells were seeded in 24-well plates (BD Falcon™). After five days of PMA-induced differentiation (0.1 µg/mL), the macrophages were treated 3 h with LPS-EB and KO (previously incubated as described). After the treatment, the culture medium was harvested and centrifuged at 500× *g* (5 min). The cells were homogenized in RIPA buffer (Cell Signaling Technology, Inc., Danvers, MA, USA) for protein quantification using the bicinchoninic acid assay (Thermo Fisher Scientific, Bonn, Germany). Human TNF-α was determined in the supernatant using the DuoSet-ELISA kit (R&D Systems Europe, Ltd., Abingdon, UK) according to the manufacturer’s instructions; 96-well NUNC MaxiSorp™ (Thermo Fisher Scientific) were used. The amount of TNF-α was normalized with the protein content.

### 4.8. Statistical Analyses

The SigmaPlot^®^-12 software (Systat Software GmbH, Erkrath, Germany) was used to carry out statistical analyses by one-way analysis of variance test (ANOVA) using Dunnett’s method appropriate for multiple comparisons versus the control group. Data are shown as mean + SEM.

## Figures and Tables

**Table 1:** Krill oil-in-water emulsion.

Ingredient	Amount/1000 mL
Glycerol	25.0 g
Krill Oil *	50.0 g
Sterile Water for Injection	1000 mL
pH	6.89 ± 0.06

* Virgin Krill Oil, LLC, Braintree, MA, USA, Lot #1107091, Key Ingredient Totals (*w*/*w*): *n*-3-FAs = 26%; PLs = 41.2%.

## References

[1] Bochsler P.N., Maddux J.M., Neilsen N.R., Slauson D.O. (1993). Differential binding of bacterial lipopolysaccharide to bovine peripheral-blood leukocytes. Inflammation.

[2] Adams J.L., Czuprynski C.J. (1990). Bacterial lipopolysaccharide induces release of tumor necrosis factor-alpha from bovine peripheral blood monocytes and alveolar macrophages in vitro. J. Leukoc. Biol..

[3] Sprague A.H., Khalil R.A. (2009). Inflammatory cytokines in vascular dysfunction and vasculardisease. Biochem. Pharmacol..

[4] Cavaillon J.M., Fitting C., Haeffner-Cavaillon N., Kirsch S.J., Warren H.S. (1990). Cytokine response by monocytes and macrophages to free and lipoprotein-bound lipopolysaccharide. Infect. Immun..

[5] Emancipator K., Csako G., Elin R.J. (1992). In vitro inactivation of bacterial endotoxin by human lipoproteins and apolipoproteins. Infect. Immunol..

[6] Eggesbo J.B., Hjermann I., Hostmark A.T., Kierulf P. (1996). LPS induced release of IL-1 beta, IL-6, IL-8 and TNF-alpha in EDTA or heparin anticoagulated whole blood from persons with high or low levels of serum HDL. Cytokine.

[7] Baumberger C., Ulevitch R.J., Dayer J.M. (1991). Modulation of endotoxic activity oflipopolysaccharide by high-density lipoprotein. Pathobiology.

[8] Wu A., Hinds C.J., Thiemermann C. (2004). High-density lipoproteins in sepsis and septic shock: Metabolism, actions, and therapeutic applications. Shock.

[9] Shor R., Wainstein J., Oz D., Boaz M., Matas Z., Fux A., Halabe A. (2007). Low serum LDL cholesterol levels and the risk of fever, sepsis, and malignancy. Ann. Clin. Lab. Sci..

[10] Calder P.C. (2006). *n*-3 polyunsaturated fatty acids, inflammation, and inflammatory diseases. Am. J. Clin. Nutr..

[11] Arterburn L.M., Hall E.B., Ojen H. (2006). Distribution, interconversion and dose response of *n*-3 fatty acids in humans. Am. J. Clin. Nutr..

[12] Nash S.M.B., Schlabach M., Nichols P.D. (2014). A nutritional-toxicological assessment of antarctic krill oil versus fish oil dietary supplements. Nutrients.

[13] Wichmann M.W., Thul P., Czarnetski H.D., Morlion B.J., Kemen M., Jauch K.W. (2007). Evaluation of clinical safety and beneficial effects of a fish oil containing lipid emulsion (Lipoplus, MLF541): Data from a prospective, randomized, multicenter trial. Crit. Care Med..

[14] Heller A.R., Rössler S., Litz R.J., Stehr S.N., Heller S.C., Koch R., Koch T. (2006). Omega-3 fatty acids improve diagnosis-related clinical outcome. Crit. Care Med..

[15] Barbosa V.M., Miles E.A., Calhau C., Lafuente E., Calder P.C. (2010). Effects of a fish oil containing lipid emulsion on plasma phospholipid fatty acids, inflammatory markers, and clinical outcomes in septic patients: A randomized, controlled clinical trial. Crit. Care.

[16] Mayer K., Fegbeutel C., Hattar K., Sibelius U., Krämer H.J., Heuer K.U., Temmesfeld-Wollbrück B., Gokorsch S., Grimminger F., Seeger W. (2003). Omega-3 vs. omega-6 lipid emulsions exert differential influence on neutrophils in septic shock patients: Impact on plasma fatty acids and lipid mediator generation. Intensive Care Med..

[17] Tsutsumi R., Horikawa Y.T., Kume K., Tanaka K., Kasai A., Kadota T., Tsutsumi Y.M. (2015). Peptide-Based Formulas With ω-3 Fatty Acids Are Protective in LPS-Mediated Sepsis. JPEN J. Parenter. Enter. Nutr..

[18] Calder P.C. (2015). Marine omega-3 fatty acids and inflammatory processes: Effects, mechanisms and clinical relevance. Biochim. Biophys. Acta.

[19] Ramsvik M.S., Bjørndal B., Bruheim I., Bohov P., Berge R.K. (2015). A Phospholipid-Protein Complex from Krill with Antioxidative and Immunomodulating Properties Reduced Plasma Triacylglycerol and Hepatic Lipogenesis in Rats. Mar. Drugs.

[20] Ghasemifard S., Turchini G.M., Sinclair A.J. (2014). Omega-3 long chain fatty acid “bioavailability”: A review of evidence and methodological considerations. Prog. Lipid Res..

[21] Cansell M., Nacka F., Combe N. (2003). Marine lipid-based liposomes increase in vivo FA bioavailability. Lipids.

[22] Bistrian B.R. (2003). Clinical aspects of essential fatty acid metabolism: Jonathan Rhoads Lecture. JPEN J. Parenter. Enter. Nutr..

[23] Wohlmuth C., Dünser M.W., Wurzinger B., Deutinger M., Ulmer H., Torgersen C., Schmittinger C.A., Grander W., Hasibeder W.R. (2010). Early fish oil supplementation and organ failure in patients with septic shock from abdominal infections: A propensitymatchedcohort study. JPEN J. Parenter. Enter. Nutr..

[24] Su G.L. (2002). Lipopolysaccharides in liver injury: Molecular mechanisms of Kupffer cell activation. Am. J. Physiol. Gastrointest. Liver Physiol..

[25] Yegenaga I., Hoste E., Van Biesen W., Vanholder R., Benoit D., Kantarci G., Dhondt A., Colardyn F., Lameire N. (2004). Clinical characteristics of patients developing ARF due to sepsis/systemic inflammatory response syndrome: Results of a prospective study. Am. J. Kidney Dis..

[26] Angus D.C., Linde-Zwirble W.T., Lidicker J., Clermont G., Carcillo J., Pinsky M.R. (2001). Epidemiology of severe sepsis in the United States: Analysis of incidence, outcome, and associated costs of care. Crit. Care Med..

[27] Annane D., Belissant E., Cavaillon J.M. (2005). Septic shock. Lancet.

[28] Thomas S., Balasubramanian K.A. (2004). Role of intestine in postsurgical complications: Involvement of free radicals. Free Radic. Biol. Med..

[29] Coquerel D., Kušíková E., Mulder P., Coëffier M., Renet S., Dechelotte P., Richard V., Thuillez C., Tamion F. (2013). Omega-3 polyunsaturated fatty acids delay the progression of endotoxic shock-induced myocardial dysfunction. Inflammation.

[30] Yates C.M., Calder P.C., Ed Rainger G. (2014). Pharmacology and therapeutics of omega-3 polyunsaturated fatty acids in chronic inflammatory disease. Pharmacol. Ther..

[31] Bonaterra G.A., Wakenhut F., Röthlein D., Wolf M., Bistrian B.R., Driscoll D., Kinscherf R. (2014). Cytoprotection by omega-3 fatty acids as a therapeutic drug vehicle when combined with nephrotoxic drugs in an intravenous emulsion: Effects on intraglomerular mesangial cells. Toxicol. Rep..

[32] Zhao Y., Joshi-Barve S., Barve S., Chen L.H. (2004). Eicosapentaenoic acid prevents LPS induced TNF-α expression by preventing NF-κB activation. J. Am. Coll. Nutr..

[33] Calder P.C. (2006). Use of fish oil in parenteral nutrition: Rationale and reality. Proc. Nutr. Soc..

[34] Trebble T., Arden N.K., Stroud M.A., Wootton S.A., Burdge G.C., Miles E.A., Ballinger A.B., Thompson R.L., Calder P.C. (2003). Inhibition of tumour necrosis factor-α and interleukin-6 production by mononuclear cells following dietary fish-oil supplementation in healthy men and response to antioxidant co-supplementation. Br. J. Nutr..

[35] Wallace F.A., Miles E.A., Calder P.C. (2003). Comparison of the effects of linseed oil and different doses of fish oil on mononuclear cell function in healthy human subjects. Br. J. Nutr..

[36] Mascioli E.A., Leader L., Flores E., Trimbo S., Bistrian B., Blackburn G. (1988). Enhanced survival to endotoxin in guinea pigs fed iv fish oil emulsion. Lipids.

[37] Kulabukhov V.V. (2008). Use of an endotoxin adsorber in the treatment of severe abdominal sepsis. Acta Anaesthesiol. Scand..

[38] Weinstock C., Ullrich H., Hohe R., Berg A., Baumstark M.W., Frey I., Northoff H., Flegel W.A. (1992). Low density lipoproteins inhibit endotoxin activation of monocytes. Arterioscler. Thromb..

[39] Gordon B.R., Parker T.S., Levine D.M., Feuerbach F., Saal S.D., Sloan B.J., Chu C., Stenzel K.H., Parrillo J.E., Rubin A.L. (2006). Neutralization of endotoxin by a phospholipid emulsion in healthy volunteers. J. Infect. Dis..

[40] Dellenger R.P., Tomayko J.F., Angus D.C., Opal S., Cupo M.A., McDermott S., Ducher A., Calandra T., Cohen J. (2009). Lipid Infusion and Patient Outcomes in Sepsis (LIPOS) Investigators. Efficacy and safety of a phospholipid emulsion (GR270773) in gram-negative severe sepsis: Results of a phase II multicenter, randomized, placebo-controlled, dose finding trial. Crit. Care.

[41] European Food Safety Authority (EFSA) (2010). Panel on Biological Hazards. Scientific opinión on fish oil for human consumption. EFSA J..

